# Data for functional TiO_2_ embedded Silicon photodetectors under varying illumination and bias conditions

**DOI:** 10.1016/j.dib.2019.104856

**Published:** 2019-11-21

**Authors:** Khushbu R. Chauhan, Dipal B. Patel

**Affiliations:** Department of Physics, Lovely Professional University, Phagwara, Punjab, 144411, India

**Keywords:** TiO_2_ nanocrystals, Responsivity, Detectivity, Noise, Sensitivity, Gain, Linear dynamic range (LDR)

## Abstract

In this data in brief (DIB) article, major photodetector (PD) characteristics of anisotype (Ag/n-TiO_2_/p-Si/Al), isotype (Ag/n-TiO_2_/n-Si/Ag) and M-S-M type (Ag/p-Si/Al) structures under reverse bias conditions (−1 to −5 V) over a broad spectral region (300–800 nm) have been presented. Critical figures of merit like current-voltage (IV), responsivity (R), detectivity (D), gain, sensitivity (S), linear dynamic range (LDR), normalized photo to dark current ratio (NPDR) and noise equivalent power (NEP) of TiO_2_ embedded Si PDs are presented in graphical forms. I–V characteristics of PDs under dark and monochromatic illuminations (365, 425, 515 and 600 nm) were acquired by using source measure unit (Kithley). Internal gain was deduced from photoresponse spectra which were recorded with the help of Potentiostat/Galvanostat (PGSTAT302N, Autolab) under monochromatic illumination at 100 Hz chopping frequency. Quantum efficiency instrument supplied by Optosolar was utilized to accurately measure the spectral responsivity and detectivity of PDs in wide spectral region (300–1100 nm). Please refer our main article [1] to understand the role of functional nanocrystalline TiO_2_ films on the performance of the photodetectors.

**Table:** Specifications Table

Subject area	*Physics*
More specific subject area	*Silicon photodetectors*
Type of data	*graph, figure, table*
How data was acquired	*Photoresponse was recorded with the help of Potentiostat/Galvanostat, Spectral responsivity and detectivity measurements were performed by using QE instrument, IV characteristics were obtained by using source measure unit embedded with monochromatic light source. At the end, all the figures of merit were calculated by using the equations as in main manuscript* [1].
Data format	*Raw and Analyzed*
Experimental factors	*A thin layer of Ti was sputtered on n-Si and p-Si followed by vacuum annealing to form functional TiO*_*2*_*/Si junctions*
Experimental features	*Three configurations of Si based photodetector were realized to compare their performance in a wide spectral region (UV-NIR)*
Data source location	*Gandhinagar, Gujarat, India*
Data accessibility	*Data is with this article*
Related research article	*K. R. Chauhan, D. B. Patel,* Functional nanocrystalline TiO_2_ thin films for UV enhanced highly responsive Silicon photodetectors, J. Alloys Compd. 792 (2019) 968–975.

**Table:** 

**Value of the Data** • The data presented in this data article is of high importance to the researchers as well as industries working towards the development of highly sensitive, responsive, ultrafast, broadband Si photodetectors. • Detailed figures of merit of three configurations of Si PDs under varying illumination and reverse bias conditions are quantitatively analyzed and graphically exemplified over broadband region. • Spectral responsivity and detectivity are the key PD parameters to be used as ready reckoner to see the effect of functional TiO_2_ film on overall performance of Si PDs. • Availability of the PDs data over a broadband range accelerates their direct integration in modern electronics and application based design of PDs can be directly chosen for the future developments.

## 1 Data

**Fig. 1:**
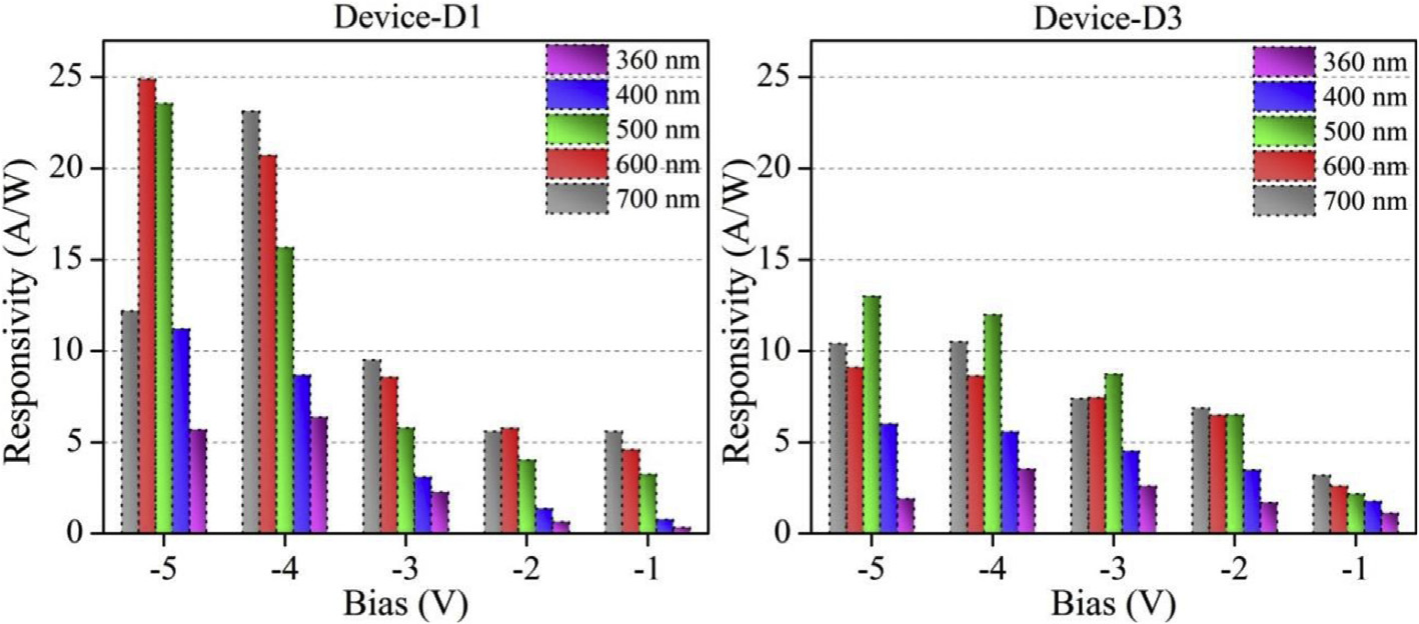
Variation in responsivity with applied bias and illumination conditions.

**Fig. 2:**
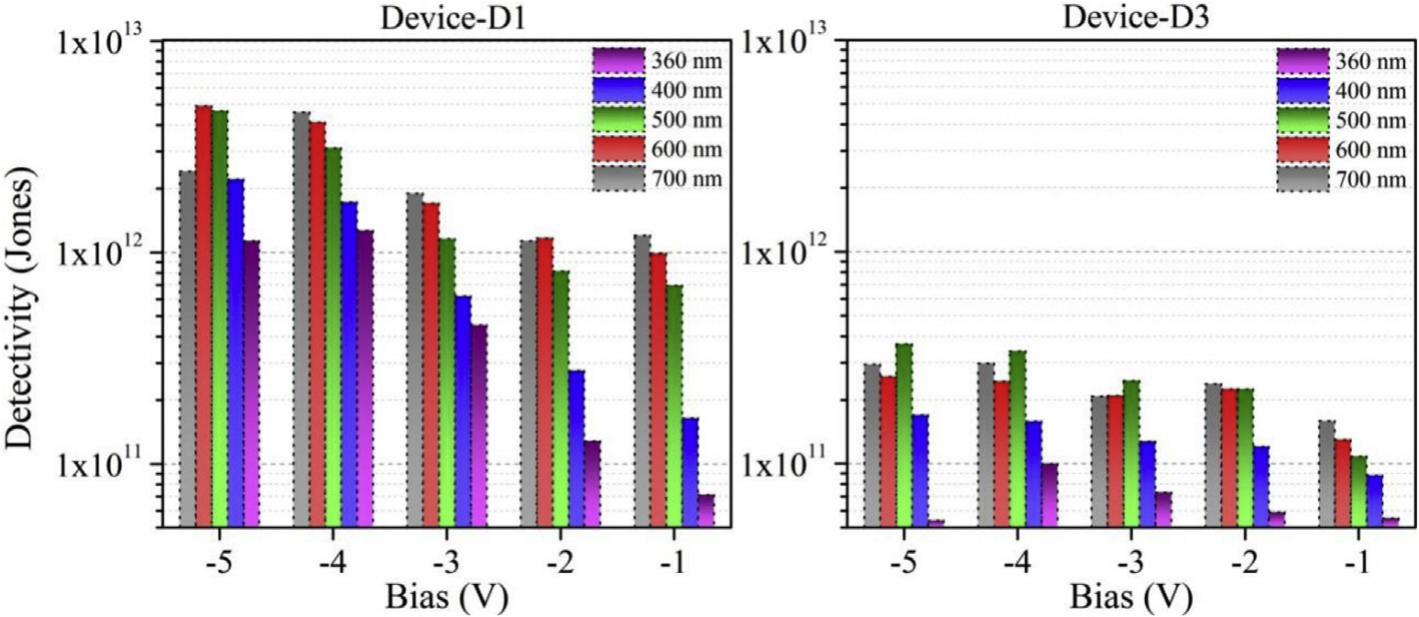
Variation in detectivity with applied bias and illumination conditions.

**Fig. 3:**
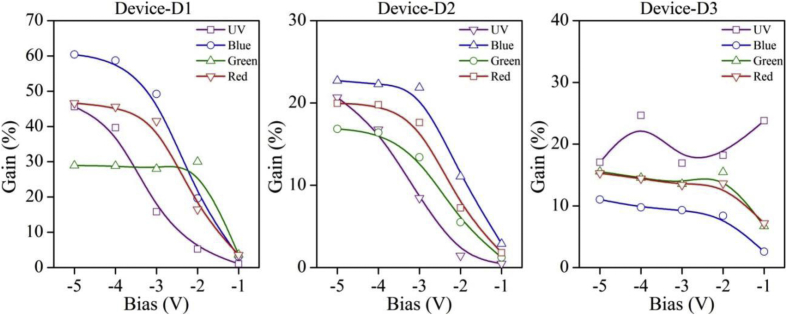
Variation in photo-gain with applied bias and illumination conditions. B-spline function has been used to show the estimated trend of the gain for each device.

**Fig. 4:**
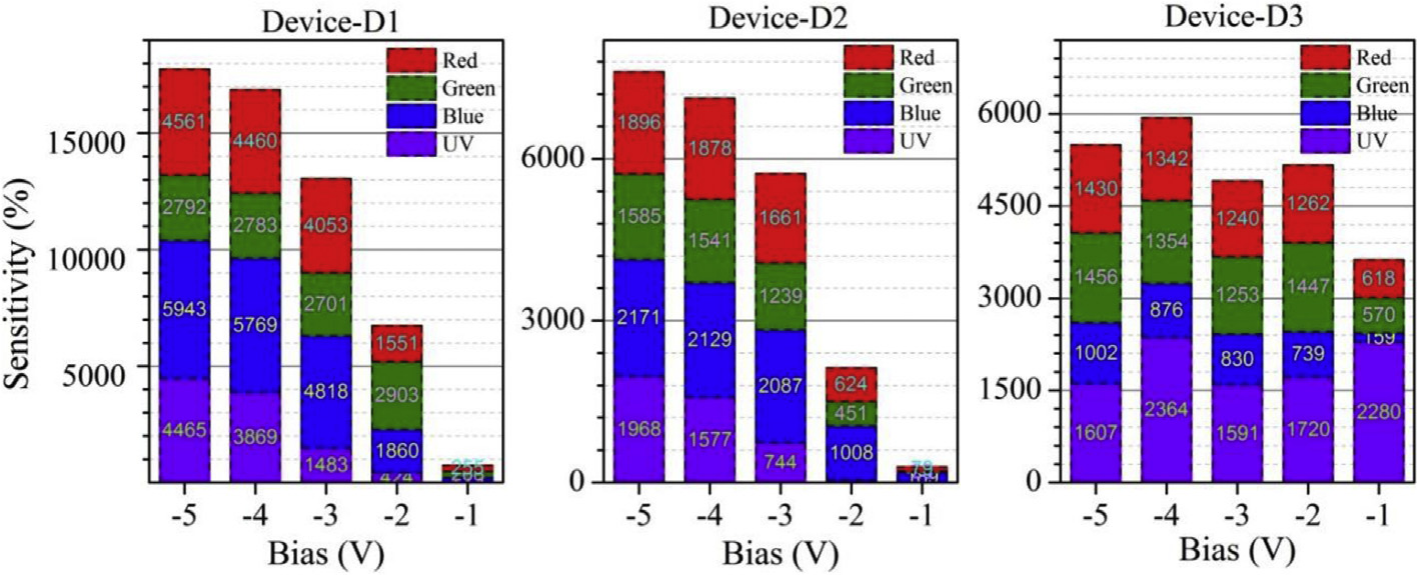
Variation in sensitivity with applied bias and illumination conditions.

**Fig. 5:**
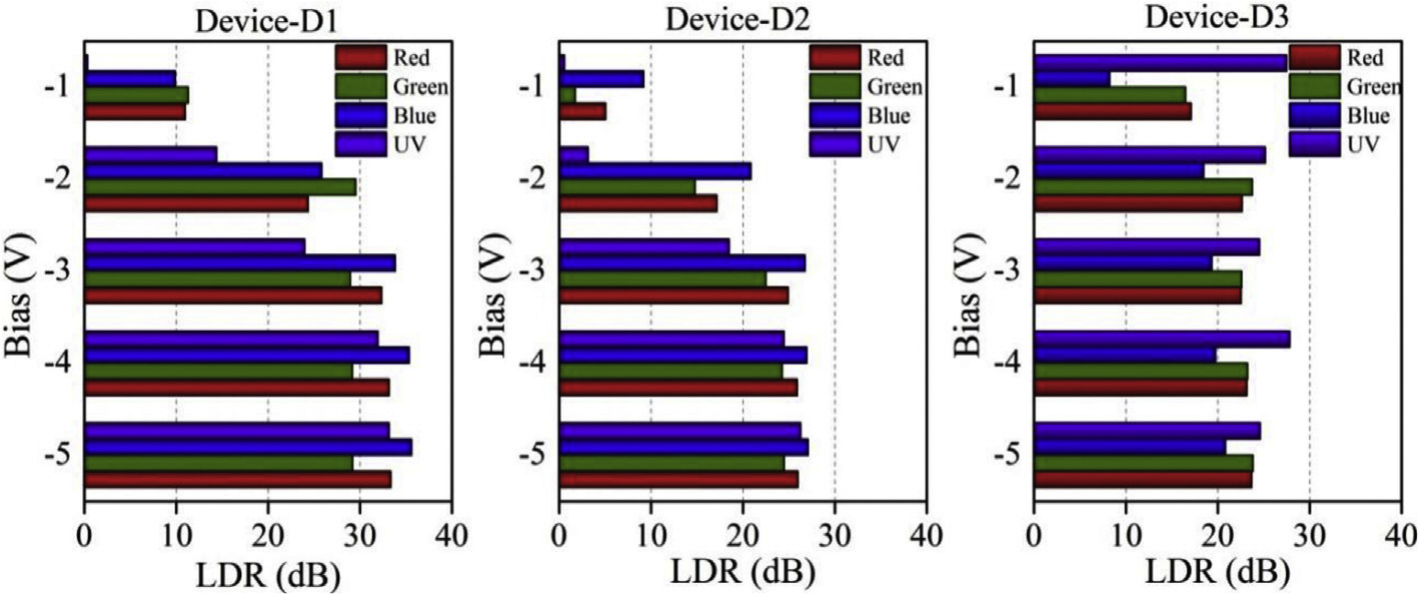
Variation in LDR with applied bias and illumination conditions.

Functioning of low electron affinity nanocrystalline TiO_2_ embedded Si PDs were studied in our recent article [1] in which Ag/n-TiO_2_/p-Si/Al anisotype junction was found to be most efficient amongst all PDs. This DIB article includes all the analyzed PD parameters which were utilized to get insight into a role of functional TiO_2_ film on the overall performance of each Si PDs. Briefly, to trace the exact contribution of a thin TiO_2_ layer, performance of Ag/n-TiO_2_/p-Si/Al was compared with Ag/p-Si/Al and hence responsivity (R) and detectivity (D) of such devices (D1 and D3) are presented in Fig. 1, Fig. 2, respectively. Fig. 1 shows the variation in responsivity (in A/W) of the PDs with varying bias under the illumination of typically selected wavelengths. Viz., 360, 400, 500, 600 and 700 nm representing UV, blue, green, red and near infrared (NIR) regions, respectively. Detectivity (in Jones) variation of such devices is shown in Fig. 2 in the form of bar charts for the mentioned lights and reverse bias conditions. Photocurrent gain for all three configurations of Si PDs are presented in Fig. 3 consecutively from left to right for the devices Ag/n-TiO_2_/p-Si/Al (D1), Ag/n-TiO_2_/n-Si/Ag (D2) and Ag/p-Si/Al (D3), respectively. Enhancement in the photocurrent against the dark current of each PD while operated in the reverse bias can be readily looked into from these graphs. Sensitivity of any PD believed to be one of the most crucial figures of merit and thus Fig. 4 includes the sensitivity data of each PD under the monochromatic illumination from broad spectral range. Fig. 5 shows very important behavior of PDs in the form of linear dynamic range which signifies the degree of linearity in PD operation against its noise. It includes LDR response of all the devices operated under reverse bias and predefined illuminating wavelengths.

**Fig. 6:**
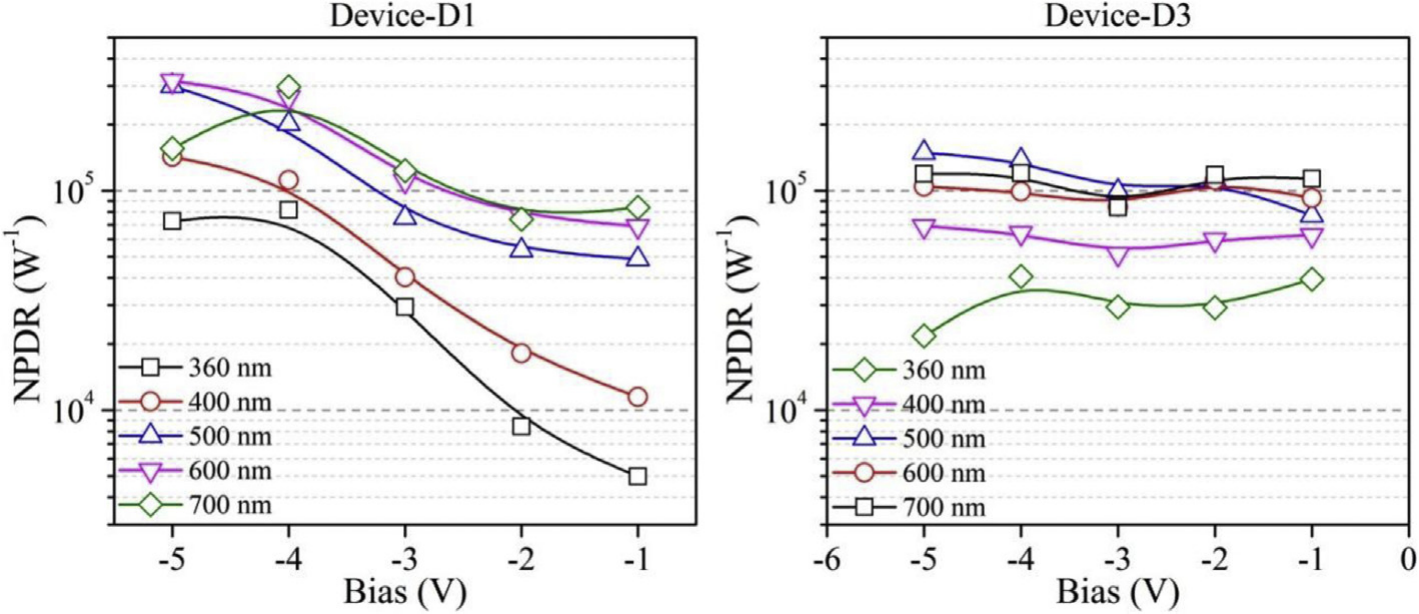
Variation in NPDR with applied bias and illumination conditions. B-spline function has been used to show the estimated trend of NPDR for each device.

**Fig. 7:**
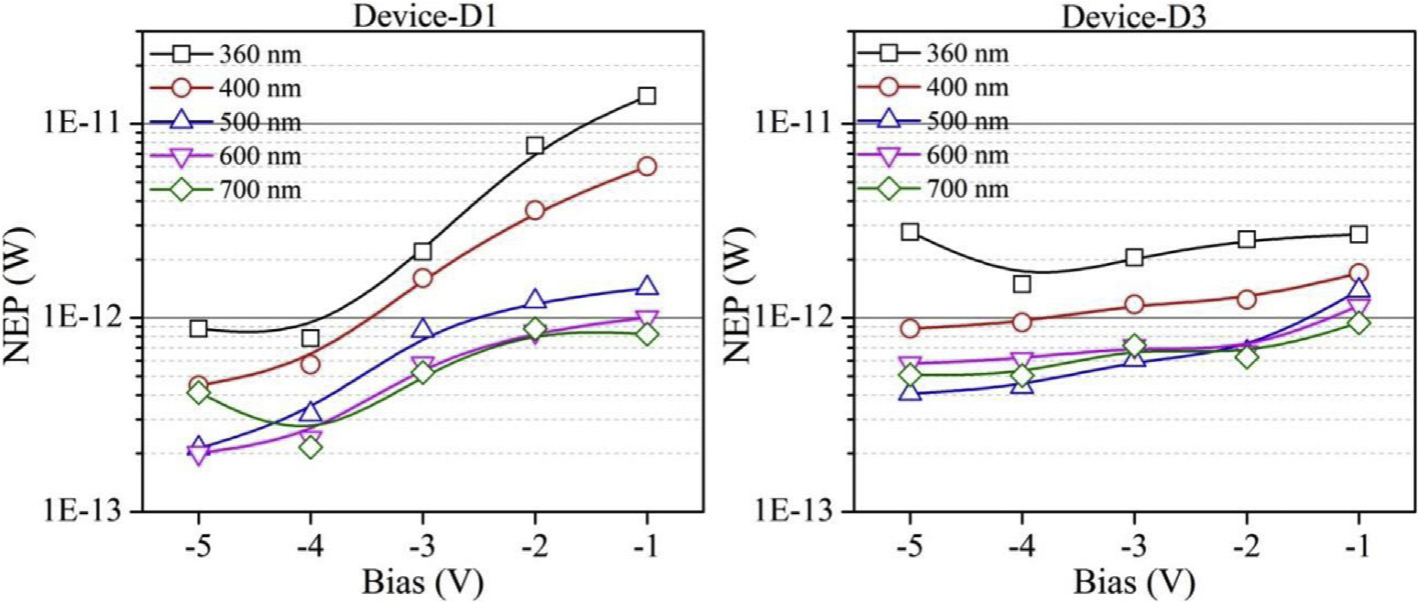
Variation in NEP with applied bias and illumination conditions. B-spline function has been used to show the estimated trend of NEP for each device.

**Fig. 8:**
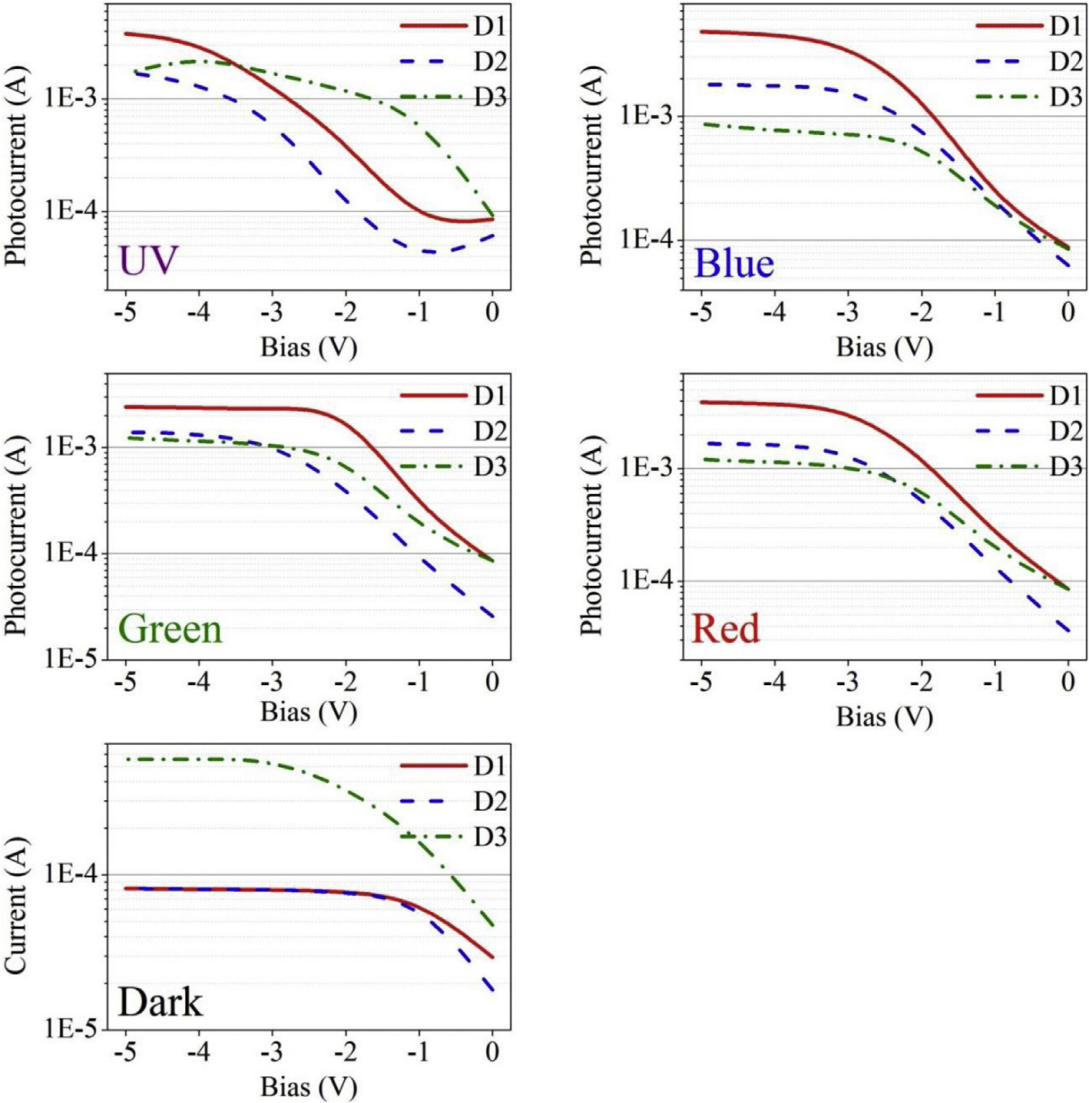
Recorded IV spectra of PDs under reverse bias upon illumination and dark conditions.

Quantitatively analyzed normalized photo to dark current ratio (NPDR) and noise equivalent power (NEP) are shown in Fig. 6, Fig. 7, respectively. Variation in NPDR and NEP with applied bias is highly important to trace out the ability of designed PD in handling the noise level and thus enabling a quicker response to the actual signal. At the end, IV characteristics of each of the PDs under varying illumination and dark conditions are shown in Fig. 8.

All the acquired raw data and analyzed figures of merit like responsivity, detectivity, gain, sensitivity, LDR, NPDR and NEP of designed PDs are given in Table 1, Table 2, Table 3, Table 4, Table 5, Table 6, Table 7, respectively.

**Table 1:** Responsivity of devices D1 and D3 under varying illumination (UV, Blue, Green, Red and NIR) and bias (−1 to −5 V) conditions.

Responsivity (A/W)										
Applied Bias (V)	Wavelength (nm)									
	360		400		500		600		700	
	D1	D3	D1	D3	D1	D3	D1	D3	D1	D3
−1	0.33	1.11	0.77	1.77	3.25	2.17	4.59	2.6	5.6	3.19
−2	0.64	1.7	1.37	3.47	4.03	6.49	5.77	6.49	5.6	6.87
−3	2.27	2.59	3.1	4.53	5.79	8.73	8.55	7.44	9.51	7.38
−4	6.36	3.54	8.68	5.59	15.67	11.98	20.71	8.64	23.13	10.49
−5	5.7	1.9	11.2	6	23.56	13	24.89	9.1	12.19	10.4

**Table 2:** Detectivity of devices D1 and D3 under varying illumination (UV, Blue, Green, Red and NIR) and bias (−1 to −5 V) conditions.

Detectivity (×10^10^ Jones)										
Applied Bias (V)	Wavelength (nm)									
	360		400		500		600		700	
	D1	D3	D1	D3	D1	D3	D1	D3	D1	D3
−1	7.14	5.54	16.4	8.81	69.7	10.8	98.6	13	120	15.9
−2	12.8	5.91	27.6	12.1	81.4	22.6	116	22.5	113	23.9
−3	45.3	7.32	62	12.8	116	24.6	171	21	190	20.8
−4	127	10	173	15.8	312	34.0	412	24.5	460	29.7
−5	113	5.39	222	17	467	36.9	493	25.8	242	29.5

**Table 3:** Photogain of devices D1, D2 and D3 under varying illumination (UV, Blue, Green and Red) and bias (−1 to −5 V) conditions.

Gain												
Applied Bias (V)	Wavelength (nm)											
	360			400			500			600		
	D1	D2	D3	D1	D2	D3	D1	D2	D3	D1	D2	D3
−1	1.04	0.46	23.80	3.13	2.89	2.59	3.68	1.23	6.70	3.55	1.79	7.18
−2	5.24	1.45	18.20	19.60	11.08	8.39	30.03	5.51	15.47	16.51	7.24	13.62
−3	15.83	8.44	16.91	49.18	21.87	9.30	28.01	13.39	13.53	41.53	17.61	13.40
−4	39.69	16.77	24.64	58.69	22.29	9.76	28.83	16.41	14.54	45.60	19.78	14.42
−5	45.65	20.68	17.07	60.43	22.71	11.02	28.92	16.85	15.56	46.61	19.96	15.30

**Table 4:** Sensitivity of devices D1, D2 and D3 under varying illumination (UV, Blue, Green and Red) and bias (−1 to −5 V) conditions.

Sensitivity (×10^2^%)												
Applied Bias (V)	Wavelength (nm)											
	360			400			500			600		
	D1	D2	D3	D1	D2	D3	D1	D2	D3	D1	D2	D3
−1	0.04	−0.54	22.80	2.13	1.89	1.59	2.68	0.23	5.70	2.55	0.79	6.18
−2	4.24	0.45	17.20	18.60	10.08	7.39	29.03	4.51	14.47	15.51	6.24	12.62
−3	14.83	7.44	15.91	48.18	20.87	8.30	27.01	12.39	12.53	40.53	16.61	12.40
−4	38.69	15.77	23.64	57.69	21.29	8.76	27.83	15.41	13.54	44.60	18.78	13.42
−5	44.65	19.68	16.07	59.43	21.71	10.02	27.92	15.85	14.56	45.61	18.96	14.30

**Table 5:** LDR of devices D1, D2 and D3 under varying illumination (UV, Blue, Green and Red) and bias (−1 to −5 V) conditions.

LDR (dB)												
Applied Bias (V)	Wavelength (nm)											
	360			400			500			600		
	D1	D2	D3	D1	D2	D3	D1	D2	D3	D1	D2	D3
−1	0.4	−6.7	27.5	9.9	9.2	8.3	11.3	1.8	16.5	11.0	5.1	17.1
−2	14.4	3.2	25.2	25.8	20.9	18.5	29.6	14.8	23.8	24.4	17.2	22.7
−3	24.0	18.5	24.6	33.8	26.8	19.4	28.9	22.5	22.6	32.4	24.9	22.5
−4	32.0	24.5	27.8	35.4	27.0	19.8	29.2	24.3	23.3	33.2	25.9	23.2
−5	33.2	26.3	24.6	35.6	27.1	20.8	29.2	24.5	23.8	33.4	26.0	23.7

**Table 6:** NPDR of devices D1 and D3 under varying illumination (UV, Blue, Green, Red and NIR) and bias (−1 to −5 V) conditions.

NPDR (×10^3^ 1/W)										
Applied Bias (V)	Wavelength (nm)									
	360		400		500		600		700	
	D1	D3	D1	D3	D1	D3	D1	D3	D1	D3
−1	4.98	39.5	11.5	62.8	48.7	77.1	68.8	92.6	83.9	113
−2	8.42	29.3	18.1	59.8	53.4	112	76.4	112	74.2	118
−3	29.5	29.5	40.3	51.4	75.3	99.2	111	84.5	124	83.8
−4	81.9	40.6	112	64.1	202	137	267	99.1	298	120
−5	72.7	21.8	143	68.8	301	149	318	104	156	119

**Table 7:** NEP of devices D1 and D3 under varying illumination (UV, Blue, Green, Red and NIR) and bias (−1 to −5 V) conditions.

NEP (×10^−13^ W)										
Applied Bias (V)	Wavelength (nm)									
	360		400		500		600		700	
	D1	D3	D1	D3	D1	D3	D1	D3	D1	D3
−1	139	27	60.4	17	14.2	13.8	10.1	11.5	8.25	9.4
−2	77.3	25.3	35.9	12.4	12.2	6.64	8.52	6.65	8.78	6.28
−3	21.9	20.5	16	11.7	8.57	6.08	5.80	7.14	5.22	7.19
−4	7.84	14.9	5.74	9.46	3.18	4.41	2.41	6.12	2.16	5.04
−5	8.78	27.8	4.47	8.8	2.13	4.06	2.01	5.8	4.11	5.08

## 2 Experimental design, materials, and methods

Monocrystalline Si wafers of p and n-type were used as substrates to fabricate PDs of the configurations discussed in the main manuscript [1]. Ohmic metal contacts on such Si wafers were obtained by sputtering thin layers of aluminum (Al) and silver (Ag), appropriately. High purity Ti (99.995% pure, Sigma Aldrich) was sputtered at constant power of 150 W and 5 mT working pressure with predefined Ar flow for 15 min. To convert Ti thin films into titanium dioxide (TiO_2_), Ti coated Silicon films were post treated in vacuum furnace at 700 °C for 10 min.
